# Epidemiology of needlestick and sharp injuries among health care workers based on records from 252 hospitals for the period 2010–2014, Poland

**DOI:** 10.1186/s12889-019-6996-6

**Published:** 2019-05-24

**Authors:** Anna Garus-Pakowska, Mariusz Górajski

**Affiliations:** 1 Department of Hygiene and Health Promotion, Medical University of Lodz, 90-752 Lodz, Poland; 2 Department of Econometrics, University of Lodz, 90-214 Lodz, Poland

**Keywords:** Healthcare workers, Needlestick injury, Sharp injury, Occupational exposures, Underreporting, Hospitals, Surveillance

## Abstract

**Background:**

Needlestick and sharp injuries (NSIs) are an important element of public health and should be closely monitored. On the other hand there are no precise Polish data on a number of the occupational NSIs. The aim of the study was to assess the failure to report injuries and then to estimate the actual number of NSIs among healthcare workers (HCWs) in Poland based on the collected data.

**Methods:**

Analysis of injury registers on the basis of 252 hospitals in Poland. Conducting 487 surveys among doctors, nurses and paramedics. Calculation of rates of injuries per 1000 workers per year (with 95% confidence intervals (CI)). The level of statistical significance was set at *p* ≤ 0.05.

**Results:**

In the study period, 9775 NSIs were registered in the hospitals. Majority of the NSIs were recorded among nurses (72.6%,*p* < 0.01). The needle was the tool responsible for the greatest number of the NSIs in all professional groups (79.5%, *p* < 0.01). The average annual NSIs rates based on hospital registers were: 16.0/1000 doctors, 20.5/1000 nurses, 16.8/1000 paramedics. Every second NSIs was not reported (45.2%). We estimated that there are probably 13,567 NSIs every year among hospital care workers in Poland.

**Conclusions:**

NSIs are a significant health problem for HCWs and should be subject to epidemiological surveillance. The purpose of the training of medical personnel should be to increase the number of injuries reported. The implementation of the epidemiological surveillance system will allow for the unification of the obtained data, which would be more comparable on the national scale as well as between different countries.

**Electronic supplementary material:**

The online version of this article (10.1186/s12889-019-6996-6) contains supplementary material, which is available to authorized users.

## Background

In 1986, the Center for Disease Control and Prevention of Infectious Diseases (CDC) provided a definition of an epidemiologic surveillance: The ongoing, systematic collection, analysis, and an interpretation of health data essential to the planning, implementation, and evaluation of public health practice, closely integrated with the timely dissemination of that data to those who require same. The final link in the surveillance chain is the application of data to a prevention and control of the diseases [1]. Performing epidemiological surveillance is expected to bring a specific benefits to the public health. The information obtained as a result of the supervision is to determine the size of a specific threats and to allow planning of the allocation of funds allocated to disease control programs. Which diseases / events will be included in the surveillance depends on the health policy of each country. These can be the diseases that affect many people. Also, less frequent events are a significant problem if their course is severe or if they raise concerns among a population. Do needlestick and other sharp injuries (NSIs) meet these conditions?

According to the CDC, in the United States the annual number of sharp injuries among the Health Care Workers (HCWs) is 385,000 [2]. In Europe, there is an average of 0.64 needlestick injuries for every health worker per year, as estimated on injuries that occurred in 2000 [3]. Sharp injuries involve a risk of disease caused by blood-borne pathogens. These microorganisms include but are not limited to HBV, HCV and HIV [3–5]. Possible diseases are a concern of medical workers [6–8]. It should be emphasized that you can prevent NSIs. Studies have shown that the use of needles with safety-engineered protection mechanisms was associated with a significantly lower NSIs rate [9–11] and the early post-exposure procedure allows you to minimize getting sick [4]. Preventive interventions have been included in specific regulations regarding safety at work. In Europe, the Council Directive 2010/32/EU, “Prevention from sharp injuries in the hospital and healthcare sector,” came in effect in 2013. Among the prevention measures it is mentioned: awareness-raising, education, elimination of unnecessary needles, safe procedures for sharps use and disposal, banning of recapping, vaccination, use of personal protective equipment, provision of safety-engineered devices, and appropriate surveillance [12].

In Poland, there is no surveillance system regarding NSIs. The current regulations in Poland require employers to keep records but only for their own needs [13]. The data is never officially transferred to the supervising institutions; therefore, there is no official data on a number of the injuries in Poland. Regarding the importance of this health problem among HCWs, it is necessary to develop effective measures for reducing occupational exposures. For these purposes, firstly, it is extremely important to present the prevalence of NSIs in Poland, and next to estimate a probable number of stings. The aims of the study were:

- A retrospective analysis of the frequency of injuries with regards to their structure based on the hospital registers,
- An estimate of the injury rates in Poland,
- The analysis of the structure of those injuries based on a questionnaire among the HCWs,
- An assessment of the underreporting,
- An attempt to estimate the actual number of injuries among HCWs in Poland based on the collected data.

## Methods

### Data collections

#### Hospital registers

The study used a questionnaire sheet in the form of a table on NSIs among HCWs, developed for the purposes of this study. The form contained the following data: the year in which the exposure took place, the type of medical tool that caused injury, the type of medical procedure and / or activity during which the HCWs were injured, profession, number of medical personnel in the hospital on December 31 of a given year. The table to be filled out has been sent electronically to all hospitals in Poland (*N* = 956, as of 31 December 2014). In addition, a telephone conversations were held with the directors of all hospitals regarding the purpose of collecting data on the exposure of HCWs to infectious material. We collected data in 2015 for the period 2010–2014. We received data for 2014 from registers maintained on the basis of the relevant Polish regulation [13], and data from previous years from different registers kept by the hospital infection control teams or other occupational safety and health (OSH) facilities in those hospitals.

#### Questionnaires

Of 252 hospitals that had sent us official register data we selected 26 (10% from 252) hospitals to where we sent a questionnaire. We did not calculate the return rate because the survey was issued electronically for volunteers. Returning the questionnaire was taken as consent to participate in the study.

Anonymous questionnaire sheets comprising of 32 questions. The questionnaire section on the exposure to an infectious material included six general questions regarding a frequency and types of contacts of the HCWs with the infectious material, both throughout their careers and during the last 12 months preceding the survey. To determine the structure of the injuries, detailed questions were also asked the respondents regarding the last incident of exposure to the infectious material occurred. The questions concerned: type of potentially infectious material, type of exposure, exposed part of the body, a kind of sharp tool, kind of the performed activity resulting in the injury and reporting the fact of the occupational exposure.

The questionnaire included questions on demographic information: gender, profession, age, seniority (in years), workplace (big city, small town/village), a number of jobs, and the subjectively perceived personal situation of the respondents, where it was possible to answer one of two answers: 1) “I feel insecure, there is the possibility of dismissal, I do not develop professionally”, and 2) “I am professionally fulfilled, I am sure of employment and a further development”. The full data collection questionnaire is found as Additional file 1 for this article.

### Statistical analysis

To assess the prevalence of the NSIs, rates of injuries were calculated per 1000 workers per year (with 95% confidence intervals (CI)), according to the following formula:

### Injury rate depending on the profession

$$ {IR}_p=\frac{n_1}{N_1}1000 $$

where:

n_1_ - number of injuries in the individual groups of workers

N_1_ - number of workers at risk of injuries

In order to verify the research hypotheses, Fisher-Snedecor tests were carried out (to establish differences between the injuries rates in each year), Pearson’s Chi^2^ test of independence (for variables: profession and tool, profession and activity) and odds ratios (OR) were calculated (i.e. assessment of the risk of an injury by a tool, while performing a specific activity and for an assessment of the risk of an injury in a specific professional group). We also perform the t-test to verify whether OR = 1. Moreover, we built linear trend model to assess the changes in NSIs rates among occupational groups. The intercept and slope of the trend lines were estimated using ordinary least square estimators.

For data from questionnaires we used nonparametric tests for the independence and correlation in order to study the existence of stochastic relationship between variables describing the general population. We decided to use three tests: Pearson’s chi-squared test and its modification for nominal variables based on V-Cramer statistics and Spearman test. Furthermore, to compare several of the dependent groups, we calculated the Kendall’s coefficient of concordance.

Based on the data from the registers and the responses from the surveys concerning the percentage of not reporting incidents, as well as the number of HCWs in Poland working in healthcare facilities, we have estimated the number of NSIs among HCWs in Poland.

Statistical analyses were performed using IBM SPSS Statistics 20 and Excel. In statistical tests, the results with *p* ≤ 0.05 were considered statistically significant.

Bioethical Committee Board of the Medical University of Lodz approved the study (Document No. RNN / 360/14 / KB of 05.13.2014).

## Results

### Registers

The filled tables with data came from 252 hospitals (return index = 26.36%), representing an average of 28,051 physicians, 64,806 nurses/midwifes and 3449 paramedics / per year. The number of exposed personnel examined in the study accounted for 33.88% of all physicians in Poland, 33.91% of nurses and 29.45% of the paramedics - working in healthcare facilities (based on data from the Ministry of Health in Poland).

In the study period, 9775 percutaneous injuries were registered in the hospitals. In our calculations, NSIs do not always add up to this number due to a missing data in selected variable categories. Majority of the NSIs were recorded among nurses (72.6%) (*p* < 0.01). The hollow-bore needle was the tool responsible for the greatest number of the NSIs among nurses and paramedics (76.8 and 70.0% respectively; (*p* < 0.0001). Most NSIs among doctors were caused by suture needles and hollow-bore-needles (each with around 35%).

The most frequent injuries occurred during surgeries and injections (29.8 and 26.5% respectively) (*p* < 0.0001) (Table 1).

**Table 1:** Sharp injuries among HCWs reported in Polish Hospitals, 2010–2014, by occupational group, used medical tools and performed activity

Variable	Medical tool						Total
	Hollow - bore needle	Suture needle	Scalpel		Cannula	Others*	
Physician injuries (n)	820	835	387		30	219	2291
within profession (%)	35.8	36.4	16.9		1.3	9.6	100.0
within tool (%)	13.0	68.7	49.1		4.9	40.5	24.2
within total (%)	8.7	8.8	4.1		0.3	2.3	24.2
Nurse and midwife injuries (n)	5267	372	398		508	316	6861
within profession (%)	76.8	5.4	5.8		7.4	4.6	100.0
within tool (%)	83.7	30.6	50.5		83.4	58.4	72.6
within total (%)	55.7	3.9	4.2		5.4	3.3	72.6
Paramedic injuries (n)	208	9	3		71	6	297
within profession (%)	70.0	3.0	1.0		23.9	2.0	100.0
within tool (%)	3.3	0.7	0.4		11.7	1.1	3.1
within total (%)	2.2	0.1	0.0		0.8	0.1	3.1
Total (n)	6295	1216	788		609	541	9449
within profession (%)	66.6	12.9	8.3		6.4	5.7	100.0
within tool (%)	100.0	100.0	100.0		100.0	100.0	100.0
within total (%)	66.6	12.9	8.3		6.4	5.7	100.0
Pearson’s Chi^2^ test	Chi^2^ = 2339.144 for *p* < 0.0001						
*Others - hook, scissors, pliers, etc.							
Variable	Performed activity						Total
	Blood collection	injection	Waste cleaning	surgery	Venipuncture	recapping	
Physician injuries (n)	130	253	58	1705	102	19	2267
Within profession (%)	5.7	11.2	2.6	75.2	4.5	0.8	100.0
Within activity (%)	8.0	10.3	5.2	61.6	9.2	9.0	24.4
Within total (%)	1.4	2.7	0.6	18.4	1.1	0.2	24.4
Nurse and midwife injuries (n)	1437	2164	984	1049	905	189	6728
Within profession (%)	21.4	32.2	14.6	15.6	13.5	2.8	100.0
Within activity (%)	88.2	87.8	89.0	37.9	81.8	89.2	72.5
Within total (%)	15.5	23.3	10.6	11.3	9.7	2.0	72.5
Paramedic injuries (n)	62	48	63	13	100	4	290
Within profession (%)	21.4	16.6	1.7	4.5	34.5	1.4	100.0
Within activity (%)	3.8	1.9	5.7	0.5	9.0	1.9	3.1
Within total (%)	0.7	0.5	0.7	0.1	1.1	0.0	3.1
Total (n)	1629	2465	1105	2767	1107	212	9285
Within profession (%)	17.5	26.5	11.9	29.8	11.9	2.3	100.0
Within activity (%)	100.0	100.0	100.0	100.0	100.0	100.0	100.0
Within total (%)	17.5	26.5	11.9	29.8	11.9	2.3	100.0
Pearson’s Chi^2^ test	Chi^2^ = 3121.685 for *p* < 0.0001						

The odds ratio of getting injured with a hollow-bore needle was 4.9 times greater for nurses than for other professional groups - OR = 4.9 (95% CI, 4.477, 5.406), *p* < 0.01 and almost 3.5 times greater for nurses “blood collection” relating activities OR = 3.395 (95%CI, 2.900, 3.975), *p* < 0.01, and “injection” OR = 3.573 (95%CI, 3.138, 4.069).

The average injury rate for all groups of the HCWs in the analyzed period was 19.0/1000 employees. The highest rates were recorded for nurses (average 20.5/1000), and in 2013 (average 19.7/1000). Only for physicians we noted a significant positive linear trend (slope = 0.920 and intercept = 13.24, t-test, *p* < 0.05) in averages of injury rate. According to the administrative division of Poland, the highest rates were recorded for Lubusz, Lublin Province and Opole Voivodships (36.9–25.8/1000), and the lowest for Lodzkie, Kuyavian-Pomeranian and the Masovian Voivodships (11.4–15.6/1000) (Table 2).

**Table 2:** Injuries rates/1000 HCWs in subsequent years and with division into the Voivodships

Variable	Physicians (95%CI)	Nurses (95%CI)	Paramedics (95%CI)	Average rate (95%CI)
Years (2010–2014)	16.0 (15.39, 16.71)	20.5 (19.98, 20.95)	16.8 (14.89, 18.78)	19.0 (18.61, 19.38)
2010	13.5 (12.16, 14.99)	20.2 (19.07, 21.29)	17.8 (13.46, 23.04)	18.1 (17.29, 19.02)
2011	15.5 (14.02, 16.99)	20.4 (19.27, 21.49)	18.5 (14.16, 23.67)	18.9 (18.01, 19.76)
2012	16.4 (14.91, 17.93)	20.5 (19.37, 21.59)	13.6 (9.95, 18.18)	18.9 (18.08, 19.82)
2013	17.5 (16.01, 19.06)	20.8 (19.75, 21.94)	17.6 (13.56, 22.39)	19.7 (18.87, 20.61)
2014	17.1 (15.65, 18.62)	20.5 (19.44, 21.58)	16.4 (12.59, 20.88)	19.2 (18.39, 20.08)
Voivodships				
Lower Silesian	19.3 (16.24, 22.77)	24.3 (22.11, 26.54)	13.4 (3.66, 33.90)	22.8 (21.05, 24.68)
Kuyavian-Pomeranian	6.6 (4.79, 8.89)	13.6 (11.78, 15.59)	15.6 (8.94, 25.20)	11.6 (10.27, 13.13)
Lublin Province	19.8 (16.45, 23.68)	29.8 (27.11, 32.65)	13.3 (7.47, 21.86)	26.3 (24.26, 28.55)
Lubusz	28.1 (20.51, 37.58)	39.9 (34.16, 46.39)	57.1 (15.79, 139.89)	36.9 (32.20, 42.14)
Lodzkie	9.0 (7.83, 10.34)	12.9 (11.93, 13.95)	4.9 (2.45, 8.75)	11.4 (10.65, 12.19)
Lesser Poland	22.2 (19.67, 24.96)	25.0 (23.14, 27.02)	12.4 (7.11, 20.06)	23.5 (22.04, 25.05)
Masovian	13.6 (12.10, 15.29)	16.4 (15.32, 17.64)	15.6 (11.03, 21.30)	15.6 (14.69, 16.53)
Opole	13.3 (7.07, 22.55)	30.8 (24.50, 38.30)	24.4 (10.59, 47.49)	25.8 (21.07, 31.34)
Subcarpathian	15.3 (12.47, 18.50)	20.0 (18.03, 22.21)	12.9 (5.93, 24.40)	18.6 (16.95, 20.33)
Podlaskie	17.7 (14.72, 21.13)	23.7 (21.40, 26.11)	21.9 (14.08, 32.41)	21.9 (20.13, 23.84)
Pomeranian	16.8 (14.41, 19.55)	22.9 (20.94, 25.02)	18.9 (13.74, 25.42)	21.0 (19.51, 22.63)
Silesian	30.4 (27.17, 33.93)	21.8 (20.03, 23.73)	41.6 (26.24, 62.29)	24.7 (23.12, 26.39)
Holy Cross	12.8 (10.08, 15.96)	21.9 (19.66, 24.31)	3.7 (0.45, 13.41)	18.2 (16.49, 19.97)
Warmian-Masurian	13.5 (10.39, 17.17)	21.9 (19.06, 24.95)	22.6 (13.22, 35.95)	19.3 (17.18, 21.60)
Greater Poland	18.5 (16.14, 21.18)	23.9 (22.10, 25.75)	25.3 (18.34, 33.88)	22.4 (21.00, 23.91)
West Pomeranian	15.6 (12.29, 19.64)	21.5 (18.86, 24.50)	31.1 (17.52, 50.81)	20.1 (17.95, 22.41)

95%CI – 95% Confidence Interval

### Questionnaires

We have received 487 questionnaires. The largest group of the respondents were paramedics with < 5 years of professional experience and nurses with a long-term experience extending > 25 years. There were no differences among doctors based on the seniority. We found no significant differences among the respondents based on the place of work (hospital emergency department, treatment unit and non-surgical ward) nor that related to the location: large city vs. a small city/village. 58% worked in 1 location, 27% were based in two locations. Table 3 presents the structure of the respondents by their gender and profession.

**Table 3:** Structure of the respondents due to variable gender and profession, *N* = 487 (%)

Sex	Profession			Total
	Physician (%)	Nurse (%)	Paramedic (%)	
Female	40 (14.4)	189 (68.2)	48 (17.3)	277 (100.0)
Male	36 (17.1)	7 (3.3)	167 (79.5)	210 (100.0)
Total	76 (15.6)	196 (40.2)	215 (44.1)	487 (100.0)

### Structure of the NSIs

During the 12 months period preceding the study, at least one contact with a potentially infectious material through damaged skin had 22.4% of doctors, 39.3% of nurses and 28.8% of paramedics (*p* < 0.01). 43.4% of physicians, 54.1% of nurses and 36.7% of paramedics (*p* < 0.01) admitted to a superficial puncture, while the deep wounds occurrence was 18.4, 25.5 and 11.2% respectively (*p* < 0.01).

Among the questionnaires received, as many as 248 respondents accurately remembered and described the last incident of an injury. On the basis of these cases we analyzed the NSIs structure. The structure of injuries according to the questionnaire is slightly different than according to the registers. Here the tools that caused the most NSIs were cannula and hollow-bore needle, and the most risky activities were venipuncture and blood collection (Table 4).

**Table 4:** The structure of NSIs among the respondents due to the tool and the performed activity, *N* = 248

Variable	*N* (%)
Medical tool	
Hollow-bore needle	74 (29.8)
Suture needle	41 (16.5)
Scalpel	25 (10.1)
Cannula	80 (32.3)
Others	28 (11.3)
Total	248 (100)
Performed activity	
Blood collection	44 (17.7)
Injection	31 (12.5)
Surgery	35 (14.1)
Venipuncture	82 (33.1)
Central puncture	8 (3.2)
Recapping	19 (7.7)
Others	29 (11.7)
Total	248 (100)

The largest number of the NSIs occurred with the use of a cannula during an intravenous injection and a hollow-bore needle during a blood collection. Majority of the injuries among nurses were caused by a hollow-bore needle (*p* < 0.01), among paramedics by cannula (*p* < 0.01) and among doctors by a suture needle (*p* < 0.01) and a scalpel (*p* < 0.05). Among the performed activities, the injuries usually were incurred among nurses during a blood collection (*p* < 0.05), among paramedics during a venipuncture (*p* < 0.01) and among physicians during performing a surgery *p* < 0.01). In 19 cases, most recapping occurred among nurses (*n* = 12, 63.2%) but these were not statistically significant differences (*p* = 0.71).

### Underreporting

To our question whether after the NSIs occurance those are being reported to a person responsible for the post-exposure proceedings, 90.3% (*n* = 440) answered yes, they report. Doctors are the least likely to do so (76.3% physicians (*n* = 58), 92.3% nurses (*n* = 181), 93.5% paramedics (*n* = 201); Chi^2^ = 20.492, *p* < 0.01).

We also asked if the respondents reported their latest incident of NSIs. Only 136 respondents gave an affirmative answer, which is only 54.8% of the total number of respondents. The other half of incidents (45.2%) have not been reported anywhere. Paramedics were the group most frequently reporting occurrences of the NSIs. However, no analyzed variable showed a statistical significance (Table 5).

**Table 5:** Sharp injuries reporting related to the question whether the last incident of the NSI was registered, by profession, *N* = 248

	NSIs Reporting		Total (%)
	No (%)	Yes (%)	
Physicians	26 (55.3)	21 (44.7)	47 (100.0)
Nurses	48 (46.6)	55 (53.4)	103 (100.0)
Paramedics	38 (38.8)	60 (61.2)	98 (100.0)
Total	112 (45.2)	136 (54.8)	248 (100.0)

### An attempt to estimate the average number of injuries with a sharp tools in polish hospitals

The injuries calculated on hospital registers in the examined period (2010–2014) came to 9775, which gives an average of 1955 NSIs a year.

Assuming that a notification of the NSIs was made by 54.8% HCWs, we estimated that 3568 NSIs should be reported in our registers annually. The sample of hospitals which transferred data represents 26.3% of all Polish hospitals. Based on the above data, we estimated that the average annual number of the NSIs among physicians, nurses and paramedics working in Polish hospitals should amount to 13,567 cases.

Thus, taking into account the underreporting, the annual injury rate should be as large as 32.4/1.000 HCWs (Table 6).

**Table 6:** Injury rates based on hospital registers and estimated injury rates, taking into account underreporting, in years 2010–2014, by profession

	Average number of HCWs in 252 hospitals tested	Average injury rate / 1000 HCWs	Average injury rate / 1000 HCWs having 45.2% of underreporting
Physicians	28,051	16.0	29.2
Nurses	64,806	20.5	37.4
Paramedics	3449	16.8	30.7
Total (∑)/ average (x̅)	∑ = 96,306	x̅=19.0	x̅=32.4

## Discussion

Many studies indicate that nurses are the most vulnerable to infectious material [14, 15]. Our research also confirmed this. According to surveys, nurses were most frequently exposed to NSIs. According to the registers, the highest NSIs rates were also among nurses. However, these are indicators lower than those obtained by Bush et al. where NSIs rates were 36 and 37/1000 nurses versus 16 and 38/1000 doctors depending on the type of reporting protocol [16]. Our rates, in which we included underreporting, are closer to these results.

More frequent wounds in nurses can be explained by the fact that nurses have the most frequent contact with patients. However, in our study the attention is drawn to the growing trend of NSIs rates among doctors in the coming years, which may indicate an increased interest in the problem of injuries in this professional group. In nurses and paramedics, NSIs rates in the 5-year period remained at a similar level.

Needlestick and sharp injuries represent an important occupational health issue in hospital HCWs. According to the CDC in the United States the annual number of injuries among the hospital staff is 385,000 [17]. The European Health Care Workers at Risk report provides data which states that over 1 million needlestick injuries are suffered by healthcare workers in Europe each year [18]. In France the reported incidence rates were 2.2 per 100 full time equivalent physicians and 7.0 per 100 nurses [19]. In Finland, approximately 100 NSIs/1000 healthcare workers per year were reported [20]. The estimates for Europe apply to a period before the introduction of safety-engineered instruments. The current EU Directive lays down that safety-engineered instruments should be introduced in all EU countries [12]. However, the European Biosafety Network reported that more than a third of injection devices and other sharps are still standard sharps [21].

There are no precise Polish data on a number of the occupational NSIs. Previous studies in Poland were based on registers of the selected, usually single hospital [22], sometimes based on registers of several hospitals in a selected region of Poland [23, 24]. A second type of research is a questionnaire survey done among various groups of the HCWs [25–27].

To the best of our knowledge, there is no publication that would combine both methods. In our opinion, compiled data from registers and surveys regarding in particular the underreporting in one study can give a real picture of the issue of the sharp injuries. Of course, the best source of data on NSIs is Exposure Prevention Information Network – EPINet, which is not implemented in Poland. It is a standardized tool for reporting occupational exposures, implemented in many countries around the world, thanks to which we obtain uniform data on NSIs [28–30].

According to Polish data, in 2013 in the sector of health care and social assistance, a total of 8982 work-related accidents were reported, in which 1400 events were as a result of contact with the sharp objects [31]. This is definitely less than the number 13,567 we estimated only among the hospital HCWs. This number, however, can be even greater because underreporting of injuries is a major issue. We set up 45.2% underreporting, which is consistent with some authors [15, 32]. But it is opposite to others where underreporting of NSIs to the workplace monitoring system was estimated to be about 60–70% [33, 34].

Differences in NSIs rates between provinces may also indicate inequalities in reporting injuries. You have to remember that most of the injuries can be prevented and early post-exposure procedures are proved to be effective, the willingness to report an injury should be obvious to every HCW. Therefore, you should constantly train personnel about the risks and protection against infections, especially that a large percentage of HCWs are afraid of a occupational infection after exposure to infectious material [35]. The actions of decision-makers in the field of workers protection against injuries should be preceded by a detailed description and analysis of NSIs made on the basis of uniform and reliable data. There was significant support in this study for an effective systematic surveillance system. Which will ensure the unification of the collected data. Unification of registers would primarily enable comparability of results at both national and international levels.

In collecting data, the authors encountered a number of limitations which are described in detail in the paper by Garus-Pakowska et al., 2018 [23]. It is worth to remember that the collected data is merely approximate; we estimated 45.2% underreporting but it is not known how many people failed to report the fact of puncture. The most important advantage of the study is that the data was collected from a large group of hospitals in Poland, which made it possible to unification the data on a national scale.

## Conclusions

In Poland, there are probably more than 13,567 NSIs every year among hospital doctors, nurses and paramedics and every second injury is not reported anywhere. The needle was the tool responsible for the greatest number of the NSIs and the most frequent injuries occurred during surgeries and injections. The obtained results should be used by Polish healthcare decision makers in order to provide guidance to improve prevention measures to focus on more at-risk and more frequent injuries. The purpose of the training of medical personnel should be to increase the number of injuries reported. NSIs are a significant health problem for HCWs and should be subject to epidemiological surveillance. The implementation of the epidemiological surveillance system will allow for the unification of the obtained data, which would be more comparable on the national scale as well as between different countries.

## Additional file

Additional file 1: English-language questionnaire. The full data collection questionnaire for this paper is added as a supplementary file. (DOCX 49 kb)

## Abbreviations

CDC: Center for Disease Control and Prevention of Infectious Diseases
Chi^2^: Pearson’s Chi^2^ test of independence
CI: Confidence interval
EU: European Union
HBV: Hepatitis B virus
HCV: Hepatitis C virus
HCWs: Healthcare workers
HIV: Human immunodeficiency virus
NSIs: Needlestick and sharp injuries
OR: Odds ratio
