# Supplementary material for: Epidemiology of needlestick and sharp injuries among health care workers based on records from 252 hospitals for the period 2010–2014, Poland
Source: BMC Public Health. 2019 May 24;19:634. doi: 10.1186/s12889-019-6996-6 (PMC6534898; doi:10.1186/s12889-019-6996-6)
Supplement: Supplementary file 1 — English-language questionnaire. The full data collection questionnaire for this paper is added as a supplementary file. (DOCX 49 kb) [file 12889_2019_6996_MOESM1_ESM.docx]

***Dear Sir or Madam,***

*In the Department of Hygiene and Epidemiology, Medical University of Lodz, we conduct a study on the exposure of workers to medical infectious material. We believe that given by Mr /Ms answers will contribute to greater interest in occupational health and safety, so that every employee feel comfortable and safe. The information gathered will also help to identify risky behavior committed consciously or unconsciously by the medical staff.*

*Please Mr / Ms to provide honest answers. We ensure that the survey is completely anonymous. Submission of a completed questionnaire means that you consent to the use of the information contained solely for scientific purposes (Act on the Protection of Personal Data of 29 August 1997, Journal of Laws No. 133, item 883). The information collected will be developed in the form of aggregated statistics.*

***Thank you for participating in the study****.*

**QUESTIONNAIRE**

**1. Whether in the course of your work, there were situations risky, threatening the safety of you? (during the last two years)**

a. yes, very often

b. yes, from time to time

c. very rarely

d. no, never

**2. What kind of situations they were?**

……………………………………………………………………………………………………

**3. How often has you in contact with patient's blood, body fluids, secretions or excretions?**

a. few / several times a day

b. a few times a week

c. several times a month

d. several times a year

e. never

**4. How often have you used personal protective equipment?**

|  | always | often | sometimes | never |
| --- | --- | --- | --- | --- |
| protective gloves |  |  |  |  |
| double pair of protective gloves |  |  |  |  |
| protective mask |  |  |  |  |
| protective goggles |  |  |  |  |
| protective clothing |  |  |  |  |

**5. What actions should be taken if you stabbed a contaminated needle?**

……………………………………………………………………………………………………………………………………………………………………………………………………………………………………………………………………………………………………………………………………………………………………………………………………………………………………………………………………………………..

**6. Have you ever removed the protective clothing (gloves, goggles etc.) to "easier" perform the operation with the patient?**

a. yes, very often

b. yes, but occasionally

c. no, never

d. I do not use the protective gloves

**7. How do you assess yours compliance with the procedures of hygiene (hand washing, donning gloves, etc)?**

a. always I comply with the procedures

b. sometimes I do not comply with the procedures

c. quite often I do not comply with the procedures

d. very often I do not comply with the procedures

e. in the ward where I work, there is no specific, implemented procedures

**8. How do you assess the compliance of hygienic procedures by your colleagues?**

a. very good/good

b. acceptably

c. bad, very bad

d. I do not care about this

**9. When you performed vaccination against hepatitis B?**

a. a year ago

b. 2 years ago

c. 2-5 years ago

d. more than 5 years ago, but I controlled the level of anti-HBS

e. more than 5 years ago, and I did not control the level of anti-HBS

f. I was not vaccinated

g. I do not remember whether I was vaccinated

**10. Do you feel you performed oral hygiene and vaccination protect workers from possible risks associated with the work?**

a. yes

b. no

c. I do not know

**11. How often do you injure yourself with a used needle or used medical tool?**

a. every day

b. a few times a week

c. several times a month

d. several times a year

e. less than several times a year

f. I have never injured

**12. Please indicate how often you have had contact with potentially infectious material (within the last year):**

|  | never | once | several times | dozen times | every day |
| --- | --- | --- | --- | --- | --- |
| through intact skin 12a | 1 | 2 | 3 | 4 | 5 |
| through non-intact skin 12b | 1 | 2 | 3 | 4 | 5 |
| transmucosally 12c | 1 | 2 | 3 | 4 | 5 |
| by splattering on the mucous membranes of the eye 12d | 1 | 2 | 3 | 4 | 5 |
| superficial puncture / cut 12e | 1 | 2 | 3 | 4 | 5 |
| stabbing / deep injury 12f | 1 | 2 | 3 | 4 | 5 |

**13. Does your workplace have special containers in which to put sharp instruments?**

a. yes, they are always available

b. yes, they are available, but there are not enough

c. there is no such containers

**14. What are you doing with a used needle? Are you throwing it in a special container or assumes you re-cap?**

a. always - immediately after use - I put into the container

b. usually I put into a container, but sometimes recap

c. always – at first – I recap

**15. If you ever put a cover on a used needle, have you ever had a case of injury?**

a. yes

b. no

c. I don’t remember

d. not applicable – I have never recap

**16. Are there (at your workplace) different containers / bags for infectious waste and non-infectious??**

a. yes, they are always available and properly marked

b. yes, they are available but unmarked

c. no such containers

**17. Have you ever thrown infectious material, along with other waste to an unmarked container / bag?**

a. yes

b. no

c. I don’t remember

**18. Do you - after cutting, stick injury,- report this to your supervisor or the person responsible for the conduct post-exposure?**

a. yes, immediately

b. yes, but only after some time

c. no, I do not see any threats

d. not because there is no result from the reports

e. no, because I do not know who should report such an event to

f. no, because the superior scolded me for using the equipment improperly

**The remaining questions relate to the LAST EXCHANGER OF EXPOSURE for infectious material which in your case occurred:**

*If you have never hurt yourself, go to question 26.*

**19. What kind of infectious material you were exposed to?:**

a. blood

b. saliva

c. others: ………………………..

**20. What part of your body was exposed?**

a. forearm / arm

b. palm

c. finger

d. face

e. eyes

f. others: ………………….

**21. What kind of exposure was this ?:**

a. through intact skin

b. through non-intact skin

c. by splattering on the mucous membranes of the eye

d. superficial puncture / cut

e. stabbing / deep injury

**22. Which tool was the cause of injury?**

a. suture neendle

b. hollow-bore needle

c. canula

d. scalpel

e. others: …………………………………

**23. During which action the injure took place?**

a. blood collection

b. venipuncture

c. central puncture

d. injection

e. recapping

f. surgery

g. others: ……………….

**24. Did you report the above event to the person responsible for keeping the register of professional exposures?**

a. yes, immediately

b. yes, but after a while

c. no, I have forgotten

d. no, I have not seen such a need

**25. What circumstances contributed to the injury?**

*(you can indicate more than one answer)*

a. stressful situation requiring urgent intervention (hemorrhage, collapse)

b. lack of team cooperation

c. I had a bad day, psycho-physical indisposition

d. rush

e. inattention

f. too many duties, too much workload

g. bad work organization

h. recapping

i. I did not follow the rules of work safety

j. during the procedure I thought about something else

k. short seniority, short/no work experience

l. unpredictable behavior of the patient

m. It is difficult to indicate the circumstances of the injury - with some kind of operations / activities exposure is simply very common

**Please also Mr / Ms to write some information about yourself:**

**26. Gender:** F M

**27. Profession:**

a. physician

b. nurse

c. paramedic

**28. Age (please give a finite number of years)**: .....................

**29. Number of work posts:**

a. 1

b. 2

c. 3 or more

**30. Work experience (years):**

a. < 5 years

b. 6-15 years

c.16-25 years

d. > 25 years

**31. Place of basic work**

1. big city
2. small city
3. vaillage

**32. What is the personal situation in your essential place of work**

1. I feel insecure, there is the possibility of dismissal, I do not develop professionally
2. I am professionally fulfilled, I am sure of employment and a further development

**Thank you for filling in the questionnaire.**
